# Comprehensive analysis of phenotypes and transcriptome characteristics reveal important contribution of chronic stress and inflammation in the pathogenesis of acne

**DOI:** 10.1042/BSR20260230

**Published:** 2026-07-24

**Authors:** Shuyun Yang, Chunyan Luan, Ling Xu, Yunjing Pu, Lu Xing, Xiaolan Li

**Affiliations:** 1 Department of Dermatology, The Second Affiliated Hospital of Kunming Medical University, Kunming, Yunnan, China; 2 Department of Dermatology, The People's Hospital of Baoshan, Baoshan, Yunnan, China

**Keywords:** acne vulgaris, chronic stress, inflammation, skin transcriptome

## Abstract

Acne vulgaris is a common chronic inflammatory skin disorder with a multifactorial pathogenesis involving genetic predisposition, hormonal regulation, microbial factors, and the cutaneous immune microenvironment. In recent years, psychosocial influences and their association with acne onset and progression have drawn increasing attention. Chronic stress may disturb immune homeostasis via neuroendocrine regulatory pathways and thereby contribute to acne pathophysiology. However, the molecular mechanisms by which chronic stress modulates acne development and exacerbation remain unclear. In the present study, we established a chronic stress-induced murine model of acne to characterize stress-related behavioral alterations, skin histopathological changes, serum corticosterone levels, and inflammatory mediators profiles in both serum and skin tissue. We further performed transcriptomic profiling to identify differentially expressed genes and to elucidate candidate regulatory pathways and molecular targets. Our findings indicate that chronic stress indeed contributes to the pathological progression of acne. However, stress hormones do not appear to directly drive inflammatory worsening. Instead, transcriptomic analysis indicated that chronic stress may play an important role in complex neuroendocrine–metabolic–immune interactions involved in acne exacerbation. These preliminary results provide experimental evidence exploring the role of chronic stress in acne pathogenesis and suggest potential targets for acne prevention and treatment.

## Introduction

Psychological stress triggers a cascade of physiological and pathological changes involving the hypothalamic–pituitary–adrenal (HPA) axis, the sympathetic nervous system, and the peripheral nervous system, all of which are closely associated with the onset and progression of various diseases [1]. Clinical observations have shown that psychological stress, anxiety, and depression can impair skin health and function, inducing or exacerbating skin disorders such as psoriasis, allergic skin diseases, acne vulgaris, hair disorders, and vitiligo. In turn, these appearance- or life-impacting skin diseases further compromise patients' emotional and psychological well-being [2]. A bidirectional interaction between the skin and the brain is increasingly recognized, giving rise to the concept of the ‘brain–skin axis’ [3]. Stress and the skin influence each other through multiple mechanisms to promote the development and progression of skin diseases; however, the precise pathogenic mechanisms remain unclear.

Acne vulgaris, the eighth most prevalent disease worldwide, primarily affects adolescents and may persist into adulthood in some individuals. In severe cases, it causes significant facial disfigurement and imposes substantial burdens on patients’ work and social lives [4]. Previous studies have demonstrated a definite correlation between acne severity and perceived stress levels, identifying psychological stress as a key risk factor for acne exacerbation [5]. Notably, the interaction between psychological factors and acne pathophysiology may be mediated through the release of neuropeptides and hormones, which activate abnormal cellular functions involved in acne development. For instance, stress-induced increases in glucocorticoids and androgens stimulate hyperactivity of the sebaceous glands, leading to increased sebum production, follicular obstruction, and amplification of cutaneous inflammatory responses. In addition, the release of stress-related neuropeptides can promote the production of proinflammatory cytokines, substance P, and lipids, thereby worsening acne [6,7]. Stress may also disrupt the skin microbiome, causing dysbiosis of commensal microorganisms, impairing skin barrier function, and activating innate immune responses that further drive inflammatory progression [8]. Recent studies have shown that psychological and emotional well-being is associated with the abundance of *Cutibacterium acnes* (*C. acnes*) in the skin, with lower stress levels and more positive emotional states correlating with higher abundance of this bacterial species [9]. Current treatments primarily focus on alleviating clinical symptoms and reducing lesion severity, without addressing underlying psychological factors. Therefore, elucidating the mechanisms by which stress exacerbates acne is crucial for breaking the vicious cycle between psychological stress and acne progression.

With the deepening of research in traditional Chinese medicine, numerous studies have confirmed that extracts or monomers from Chinese herbs have the potential to treat inflammatory skin diseases. For example, triptolide derivatives can specifically target the NLRP3 inflammasome and inhibit downstream inflammatory responses, which may serve as a potential strategy for acne treatment [10]. Sappanone A, a monomer isolated from *Caesalpinia sappan*, exhibits significant anti-inflammatory and immunomodulatory properties, presenting the advantages of high efficacy and low toxicity in the treatment of psoriasis [11]. Other monomers (such as resveratrol, curcumin, and epigallocatechin gallate) have been demonstrated in animal models and clinical trials to regulate the Th17/IL-17 axis, inhibit keratinization, alleviate perifollicular inflammatory responses and follicular duct obstruction [12,13]. Meanwhile, their antioxidant effects can also improve the oxidative stress microenvironment of the skin under pressure. Additionally, studies have indicated that the kidney - tonifying formula EXD and its specific chemical markers exert antidepressant effects by regulating monoamine neurotransmitter levels, inhibiting neuroinflammation, enhancing synaptic plasticity, providing neuroprotection, and modulating the HPA axis [14]. Compared with chemical drugs, traditional Chinese medicine has advantages in reducing dependence and lowering recurrence rates. Moreover, it acts on a larger number of targets simultaneously, making it a promising and ideal intervention for the regulatory network of the ‘brain–skin axis.’ However, there is currently a lack of research evidence regarding gene expression and molecular mechanisms.

In the present study, we employed a restraint stress model to simulate chronic psychological stress combined with a classical mouse model of acne vulgaris, together with high-throughput RNA sequencing and phenotypic validation, to analyze regulatory pathways and differentially expressed genes (DEGs) associated with acne progression under stress conditions. By integrating molecular and phenotypic data, the present study aims to provide experimental evidence for the role of chronic stress in acne pathogenesis and to identify potential molecular targets for future investigation.

## Materials and methods

### Animals

Male C57BL/6J mice (6–8 weeks old) were obtained from the Laboratory Animal Center of Kunming Medical University. All animal procedures were reviewed and approved by the Animal Welfare Ethics Review Committee of the Second Affiliated Hospital of Kunming Medical University (approval number: kyfey2025027). Mice were housed under specific pathogen-free conditions with a 12 h light/dark cycle, an ambient temperature of 22°C–24°C, a humidity of 50%–60%, and free access to food and water. Each mouse was tail-marked for individual identification. Mice were randomly divided into four groups (*n* = 14 per group): normal control (Control), stress-only (Pressure), acne model (Acne), and stress-exposed acne (Acne_Pressure). Among these 14 mice, ears were allocated as follows: four mice per group were used for transcriptome sequencing, four for hematoxylin–eosin (H&E) staining, and six for ear tissue enzyme-linked immunosorbent assay (ELISA). Body weight was recorded daily. At the study endpoint, the 14 mice in each group underwent behavioral testing, after which blood was collected for serum ELISA. All mice were euthanized by cervical dislocation under isoflurane anesthesia (1.5% in oxygen).

### Acne model and restraint stress

All mice were acclimated for three days prior to the start of experiments and received a daily topical application of synthetic sebum (20 μl per ear; Biochemazone, Cat# BZ328, composition: 17% fatty acids, 44.7% triglycerides, 25% jojoba oil-based wax monoesters, 12.4% squalene) to the left ear from the start of the experiment until tissue collection. The sebum was applied using a micropipette to ensure uniform coverage and was administered at the same time each day. Mice in the Acne and Acne_Pressure groups received an intradermal injection of 20 μl live *C. acnes* suspension (1 × 10^9^ CFU/ml) into the left ear, whereas mice in the Control and Pressure groups received an equal volume of PBS in the left ear (Days 4, 6, 8, 10, 12). The right ear of all mice was injected with 20 μl PBS and served as a negative control. Mice in the Pressure and Acne_Pressure groups were additionally subjected to restraint stress for 6 h per day in well-ventilated restraint tubes (Days 7–13). This 6-h daily restraint protocol is a well-established paradigm for modeling chronic psychological stress in rodents. During restraint, mice had no access to food or water.

### Behavioral tests

All behavioral tests were performed on Days 14–16 of the experiment. The testing order was open field test, elevated plus maze, and Morris water maze, with 24 h between tests. All experiments were conducted in a quiet room under dim lighting, and apparatuses were cleaned and disinfected before each trial.

Open field test: Mice were placed in an open square arena divided into a central zone and a peripheral zone. Each mouse was placed in the center and allowed to explore freely for 5 min while being recorded by a video-tracking system. The main outcome measures included total distance traveled (locomotor activity) and the time spent in and number of entries into the central zone (anxiety indices).

Elevated plus maze test: The apparatus consisted of two open arms and two closed arms, elevated 70 cm above the floor. Mice were placed in the central intersection facing an open arm, and behavior was recorded for 5 min. Time spent in the open arms, number of entries into the open arms, and total arm entries were analyzed.

Morris water maze test: The apparatus consisted of a circular pool divided into four quadrants, with a hidden platform submerged 1 cm below the water surface in one quadrant. Visual cues were placed around the pool. During training, mice were released from different quadrants and allowed up to 60 s to locate the hidden platform and remain on it for 15 s. If a mouse failed to find the platform within 60 s, it was guided to the platform and allowed to remain there for the same duration. During the probe trial, the platform was removed, and mice were released from a random starting point. Escape latency, number of crossings over the former platform location, and percentage of time spent in the target quadrant within 60 s were recorded.

### Hematoxylin–eosin staining

Ear tissues from mice in each group (*n* = 4 per group) were fixed overnight in 4% paraformaldehyde (Guangfu, CNAB035-Q), dehydrated through a graded ethanol series (Sichuan Xilong, CAS 64-17-5), cleared in xylene (Sichuan Xilong, CAS 1330-20-7), infiltrated with paraffin, and embedded in paraffin blocks. Tissues were sectioned at 3 μm. Sections were stained with hematoxylin (Servicebio, G1004) and eosin (Solarbio, G1108) and mounted with neutral resin (Sinopharm, 10004160). Representative areas were selected and examined under a light microscope (Nikon Eclipse Ci-L).

### Transcriptome sequencing analysis

Total RNA was extracted, and library construction and high-throughput RNA sequencing were performed by LC Bio Technology using the illumina Novaseq™ 6000. DEGs were identified using edgeR (v3.40.2) with the cutoff criteria of |log_2_ fold change| >1 and adjusted *P*-value <0.05. Gene Ontology (GO) and KEGG enrichment analyses and GSEA were performed for the following comparisons: Acne versus Control, Acne_Pressure versus Acne, Pressure versus Control, and Acne_Pressure versus Pressure.

### Enzyme-linked immunosorbent assay

The following kits were used: Mouse Corticosterone ELISA Kit (MEIMIAN, MM-0565M1), Mouse IL-1 ELISA Kit (MEIMIAN, MM-0039M1), Mouse IL-6 ELISA Kit (COIBO, CB10187-Mu), Mouse IL-8 ELISA Kit (MEIMIAN, MM-43835M1), and Mouse TNF-α ELISA Kit (MEIMIAN, MM-0132M2). Cell Counting Kit-8 (Elabscience, E-CK-A362), Human IL-8 ELISA Kit (MEIMIAN, MM-1558H1), Human TNF-α ELISA Kit (MEIMIAN, MM-0122H1). All standards and samples were diluted according to the manufacturers’ instructions and added to the plates, followed by incubation at 37°C. After washing the plates five times with wash buffer, enzyme conjugate was added, followed by a second incubation and washing step. Substrates A and B were then added and incubated in the dark for 10 min for color development, and the reaction was finally stopped with a stop solution. Absorbance was measured using a microplate reader. For *in vitro* experiments, cytokine concentrations in cell culture supernatants were normalized to total protein content in the corresponding cell lysates.

### Cell culture and treatments

HaCaT human-immortalized keratinocytes were cultured in complete DMEM medium (10% FBS and 1% penicillin–streptomycin) at 37°C with 5% CO_2_. For experiments, cells were divided into the following groups: a normal control group cultured under standard conditions; a model group treated with heat-inactivated *C. acnes* at a multiplicity of infection of 1:100; and model cells treated with cortisol at concentrations of 10, 100, 500, or 1000 nM. The cortisol concentration range was selected based on human physiological serum cortisol levels (normal morning range: ∼138–690 nM) and stress-induced levels reported in the literature (1000 nM corresponds to severe/chronic stress conditions). After treatment, culture supernatants were collected for cytokine measurement, and cells were lysed for total protein quantification.

### CCK-8 assay

Cells were seeded into 96-well plates at 100 μl per well and cultured for 24 h. Wells with uniform cell density were selected for grouping and treatment, and cells were cultured for 0, 12, 24, 36, 48, 60, and 72 h. At each time point, 10 μl of CCK-8 reagent was added to each well. After incubation for 2 h at 37°C, absorbance was measured at 450 nm using a microplate reader. Each experiment was performed with three independent biological replicates.

### Statistical analysis

Statistical analysis was performed using GraphPad Prism. All data are presented as mean ± SEM. For the animal experiments, data were analyzed by two-way analysis of variance (ANOVA), followed by the Bonferroni post hoc test for multiple comparisons. For the *in vitro* experiments, comparisons among groups were performed using one-way ANOVA followed by Tukey’s post hoc test. A *P*-value <0.05 was considered statistically significant. Sample sizes (*n*) for each experiment are indicated in the corresponding figure legends.

## Results

### Stress-induced behavioral changes and skin pathology in acne mice

**Figure 1:**
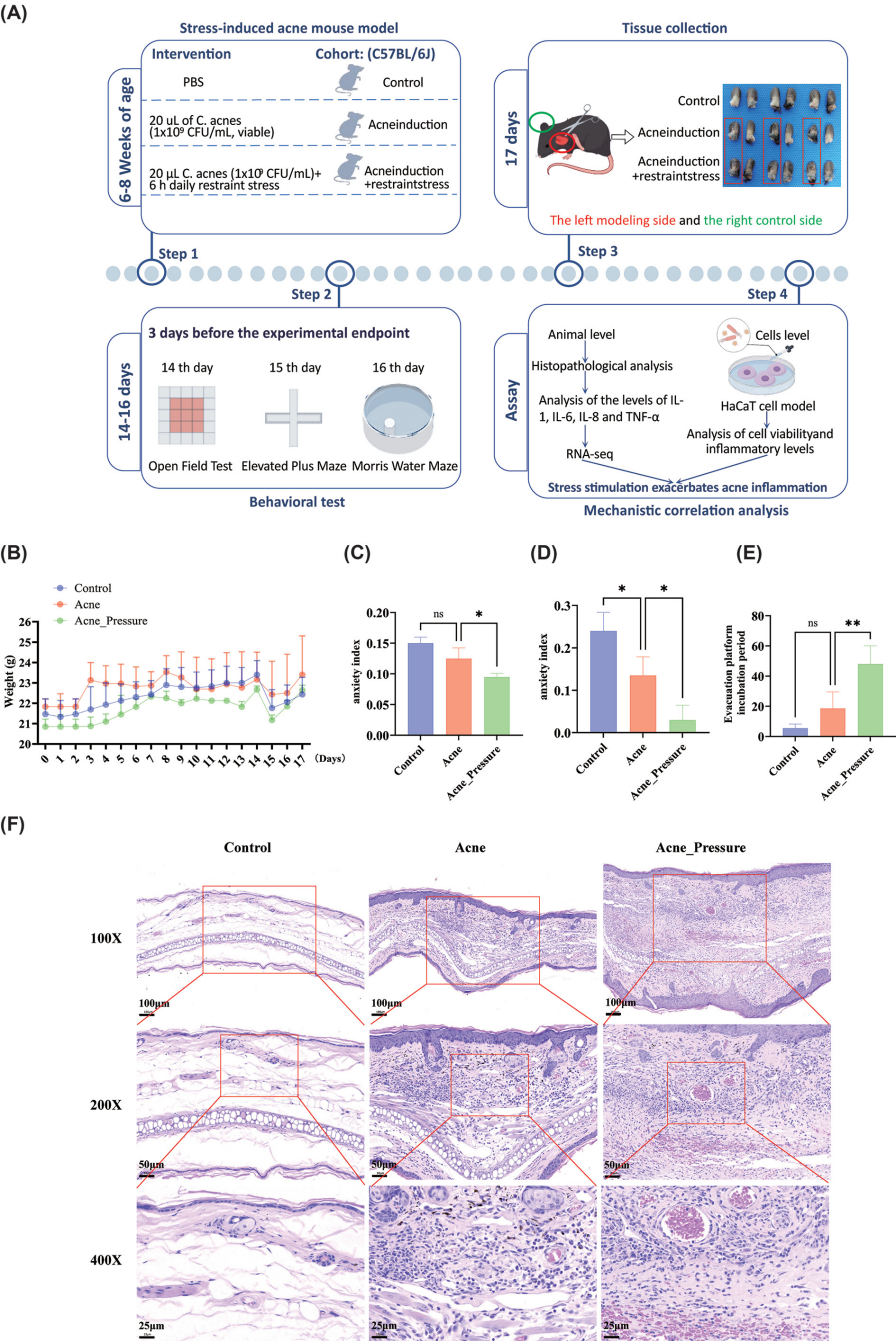
Stress exposure affects mouse behavior and exacerbates histopathological changes in acne mice (**A**) Schematic diagram of the experimental procedure. (**B**) Body weight measurements of mice in each group during the experiment (*n* = 14 per group). (**C**) Open field test results (*n* = 14 per group). (**D**) Elevated plus maze results (*n* = 14 per group). (**E**) Escape latency in the Morris water maze (*n* = 14 per group). (**F**) H&E staining showing histopathological changes in ear tissues; scale bars: 100 μm at 100×, 50 μm at 200×, and 25 μm at 400× (*n* = 4 per group). All data are presented as mean ± SEM. Data were analyzed by two-way ANOVA followed by Tukey’s post hoc test. *P<0.05, **P<0.01; ns, not significant.

To investigate whether stress promotes acne progression and its potential mechanisms, a mouse acne model was established by intradermal injection of *C. acnes* into the ears of C57BL/6J mice. During model establishment and behavioral testing, ear features of all groups were photographed, and at the experimental endpoint, left and right ear tissues were collected for analysis (Figure 1A). Gross observation revealed that the ears of control mice appeared smooth and intact, indicating healthy skin. In the acne model group (Acne), local erythema, rough epidermis, and signs of inflammation were observed in the ear tissue. In the stress-exposed acne group (Acne_Pressure), ear erythema was more pronounced, with visible tissue damage and epidermal roughness, indicating aggravated disease progression in this group. Daily body weight measurements showed a downward trend in the Acne_Pressure group (Figure 1B). Behavioral tests revealed that the Acne_Pressure group showed a pronounced decrease in anxiety index and a significantly prolonged escape latency in the water maze (Figure 1C–E and Supplementary Figure S1A–C), suggesting the successful establishment of a chronic stress model in acne mice.

Histopathological analysis of left ear tissues using H&E staining (Figure 1F) revealed that control mice had intact epidermal layers and clear dermal structures, with no obvious inflammatory cell infiltration, edema, or structural disruption, consistent with normal skin morphology. The Acne group exhibited mild epidermal thickening and a small number of inflammatory cells in the dermis. In the Acne_Pressure group, epidermal thickening was more pronounced, dermal inflammatory infiltration was more extensive and dense, and localized tissue swelling and pathological structural changes were evident. These results demonstrate that chronic stress can promote the exacerbation of acne inflammation and pathological tissue changes.

### Stress-induced behavioral changes and skin pathology in acne mice

**Figure 2:**
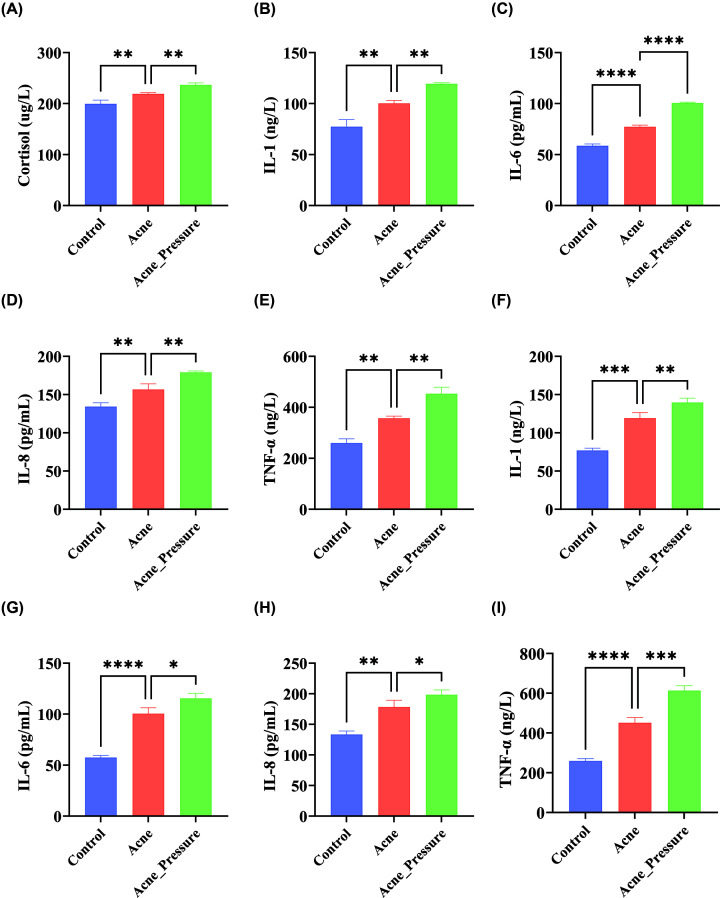
Stress exposure increases corticosterone release and elevates systemic and local inflammatory levels (**A**) ELISA measurement of corticosterone levels in mouse serum (n = 14 per group). (**B–E**) ELISA measurement of IL-1, IL-6, IL-8, and TNF-α levels in mouse serum (*n* = 14 per group). (**F–I**) ELISA measurement of IL-1, IL-6, IL-8, and TNF-α levels in left ear tissues of mice (*n* = 6 per group). All data are presented as mean ± SEM. Data were analyzed by two-way ANOVA followed by Tukey’s post hoc test. *P<0.05, **P<0.01, ***P<0.001, ****P<0.0001; ns, not significant.

Extensive studies have shown that stress can activate the HPA axis, mediating the release of cortisol and triggering widespread physiological and pathological effects [1]. We measured serum corticosterone levels by ELISA and found that stress exposure led to a significant increase in acne mice (Figure 2A). To further assess systemic and local inflammation levels, we measured proinflammatory cytokines IL-1, IL-6, IL-8, and TNF-α in serum and ear tissues (Figure 2B–I). Compared with controls, Acne mice exhibited significant up-regulation of these inflammatory factors in both serum and ear tissues, and stress exposure further enhanced their expression. These results indicate that stress amplifies inflammatory cascades in acne mice by up-regulating proinflammatory cytokine levels systemically and locally, thereby aggravating pathological damage in acne lesions. Moreover, stress stimulates abnormal systemic release of glucocorticoids. Whether the elevated glucocorticoid levels directly contribute to the exacerbation of acne inflammation remains to be further investigated.

### Effects of cortisol on *C. acnes*-induced inflammatory responses in HaCaT cells

Keratinocytes, as one of the primary host cells interacting with *C. acnes*, are active immune sentinel cells that express pattern recognition receptors such as TLR2 and TLR4 that can directly recognize specific components of *C. acnes*, leading to the release of large amounts of proinflammatory factors, thereby directly promoting inflammation and affecting keratinization [15]. It has also been demonstrated that keratinocytes express high levels of glucocorticoid receptor and mineralocorticoid receptor. When locally synthesized or systemic cortisol binds to these receptors, a complex forms that translocates into the nucleus, where it acts as a transcription factor to activate or repress the expression of specific genes, thus regulating cellular physiological activities [16].

**Figure 3:**
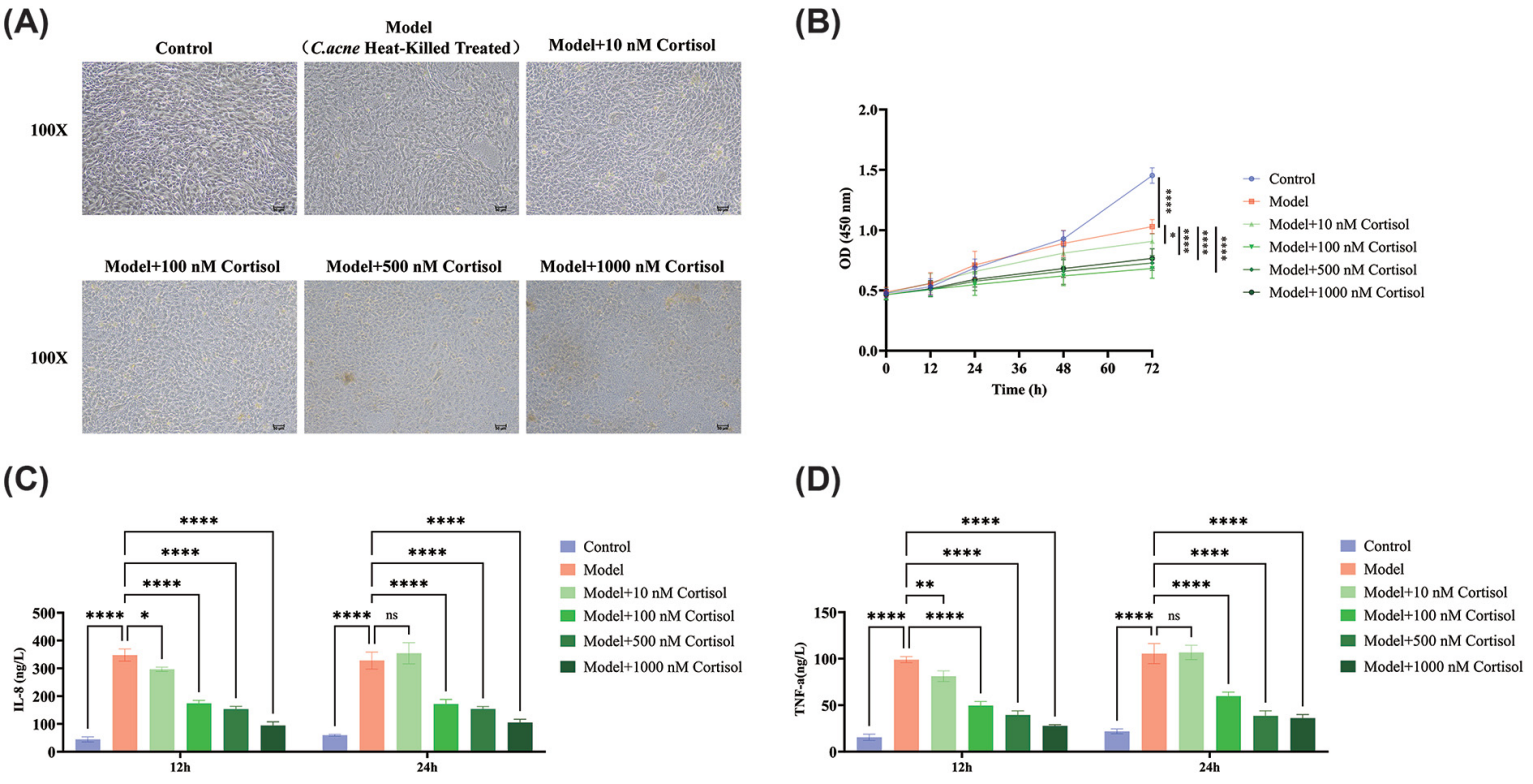
*In vitro* validation of the relationship between cortisol and acne-related inflammation (**A**) Representative images of HaCaT cells under normal culture conditions, in the acne model group (treated with heat-killed *C. acnes*), and in cortisol intervention groups at different concentrations; magnification 100×, scale bar = 50 μm (*n* = 3). (**B**) CCK-8 assay results showing cell proliferation activity (*n* = 3). (**C,D**) ELISA measurement of IL-8 and TNF-α levels in cell culture supernatants (*n* = 3). All data are presented as mean ± SEM. Data were analyzed by one-way ANOVA followed by Bonferroni post hoc test. *P<0.05, **P<0.01, ***P<0.001, ****P<0.0001; ns, not significant.

To investigate the effects of cortisol at the cellular level, we used HaCaT human immortalized keratinocytes. A model group and cortisol intervention groups (10, 100, 500, and 1000 nM) were established to analyze cell viability and inflammatory changes. Representative cell culture images are shown in Figure 3A: control cells exhibited healthy morphology and uniform distribution; cells in the acne model group (heat-inactivated *C. acnes*) showed slight morphological changes; with increasing cortisol concentrations, cell density gradually decreased and cell condition visibly worsened. CCK-8 results showed no significant changes in cell proliferation within 24 h among the control, model, and intervention groups. However, at 72 h, cell proliferation was significantly reduced in the model group compared with the control group, and cell viability decreased further with increasing cortisol concentrations (Figure 3B). Inflammatory factor analysis showed that IL-8 and TNF-α levels were significantly elevated in both the model and intervention groups compared with the control. Interestingly, with increasing cortisol concentrations, inflammatory factor levels in the culture supernatant decreased. Notably, at 12 h, treatment with 10 nM cortisol significantly reduced cytokine levels, whereas at 24 h this anti-inflammatory effect was no longer detected. In contrast, higher cortisol concentrations (100–1000 nM) significantly decreased cytokine levels at both 12 and 24 h (Figure 3C,D). These results differ from the *in vivo* findings, suggesting that cortisol may not directly drive the progression of inflammation. Instead, it likely regulates acne exacerbation through a series of complex mechanistic pathways.

### Transcriptomic analysis of pathways underlying stress-exacerbated acne progression

To further investigate the molecular mechanisms by which stress promotes acne, transcriptome sequencing was performed on the established mouse models. Comparisons were conducted for Acne versus Control, Acne_Pressure versus Acne and Acne_Pressure versus Pressure. First, volcano plots and hierarchical clustering heatmaps illustrated gene expression differences among the groups (Supplementary Figure S2A–F). GO enrichment analysis was then applied to identify cellular functions associated with DEGs. For Acne versus Control, DEGs were primarily enriched in extracellular regions, keratinization, cytokine regulation, and inflammatory responses (Supplementary Figure S2G). In Acne_Pressure versus Acne, DEGs were predominantly associated with keratinization, innate immune-related functions, extracellular space, and intermediate filament (Supplementary Figure S2H). Acne_Pressure versus Pressure encompassed bacterial defense and inflammatory responses (Supplementary Figure S2I). KEGG pathway enrichment analysis (Supplementary Figure S2J–L) revealed that DEGs were mainly enriched in inflammation-related pathways, including IL-17 signaling, cytokine–cytokine receptor interaction, chemokine signaling, and lipid metabolism pathways. Immune-related pathways, such as Toll-like receptor and NOD-like receptor signaling, were also involved.

**Figure 4:**
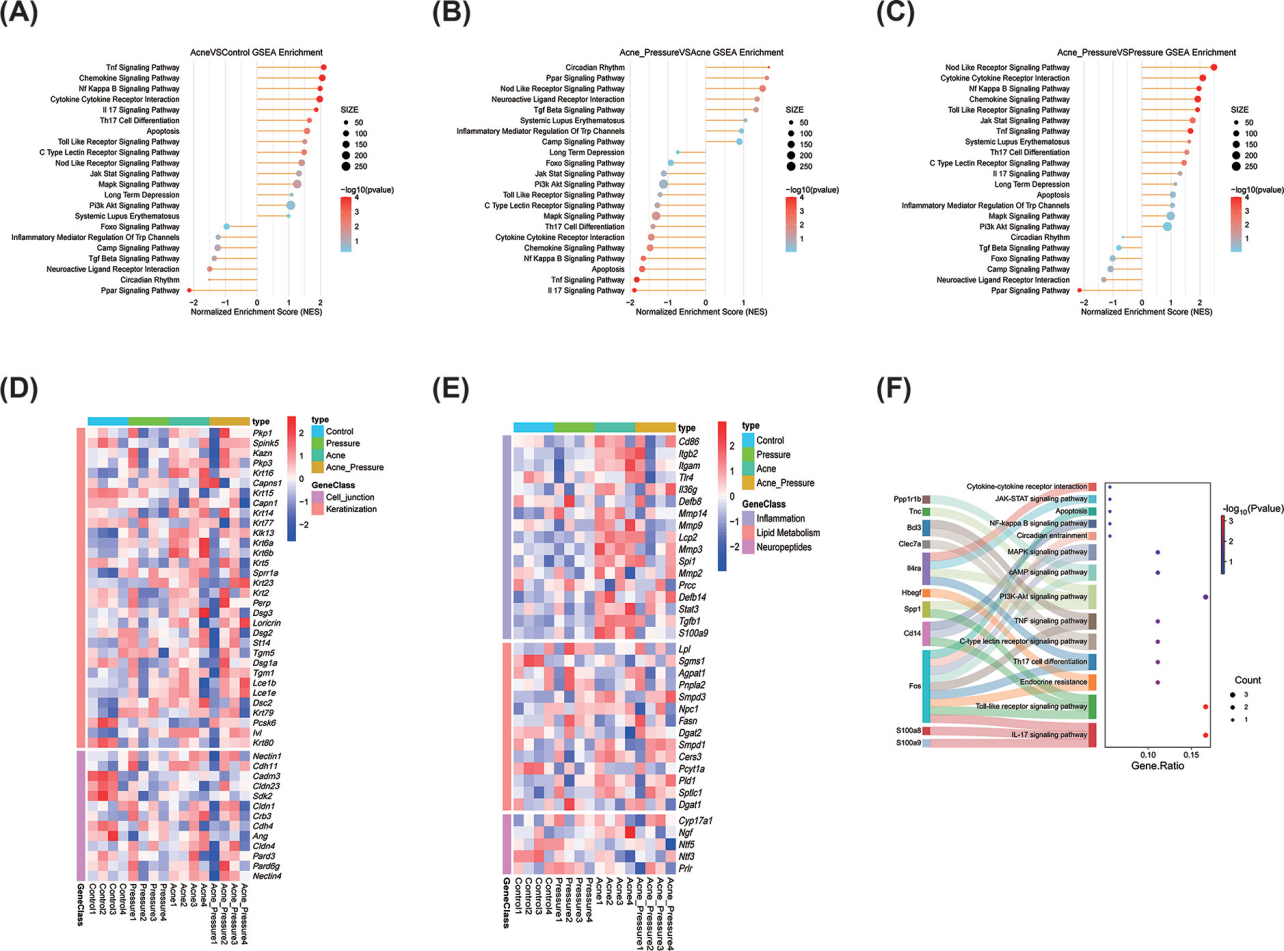
Transcriptomic analysis reveals potential pathways underlying stress-exacerbated acne progression (**A**) GSEA pathway enrichment analysis for Acne versus Control (*n* = 4 per group). (**B**) GSEA pathway enrichment analysis for Acne_Pressure versus Acne (*n* = 4 per group). (**C**) GSEA pathway enrichment analysis for Acne_Pressure versus Pressure (*n* = 4 per group). (**D**) Heatmap of gene expression patterns related to cell junctions and keratinization (*n* = 4 per group). (**E**) Heatmap of gene expression patterns related to inflammation, lipid metabolism, and neuroendocrine regulation (*n* = 4 per group). (**F**) Pathway enrichment analysis of DEGs (*n* = 4 per group).

Through GSEA pathway analyses of Acne versus Control, Acne_Pressure versus Acne, and Acne_Pressure versus Pressure, the enriched pathways were mainly involved in inflammation, lipid metabolism, and neuroregulation. Results showed that inflammatory signaling pathways and apoptotic pathways were significantly activated in the Acne group (Figure 4A), indicating that acne development is closely linked to inflammation and accompanied by cell apoptosis, consistent with previous studies [17,18]. However, lipid metabolism and neuroregulatory pathways were not significantly enriched in the Acne group. In the Acne_Pressure group and Acne group, lipid metabolism-related pathways were significantly activated, while pathways associated with neuroregulatory processes exhibited a trend toward enrichment but did not reach statistical significance (Figure 4B). Additionally, to investigate the independent effect of stress, a comparison was performed between the Pressure and Control groups. Although the number of DEGs was limited, GSEA pathway enrichment analysis revealed that the PPAR signaling pathway, a key regulator of lipid metabolism, was significantly enriched under stress alone, suggesting that stress may induce transcriptional reprogramming at lipid metabolic level (Supplementary Figure S3). Taken together with the Pressure versus Control findings, this suggests that the activation of lipid metabolic pathways observed in the Acne_Pressure group may primarily originate from stress itself rather than from acne pathology. Notably, analysis of the Acne_Pressure versus Pressure showed that the differentially enriched pathways remained mainly associated with inflammatory regulation (Figure 4C). Collectively, these pathway enrichment analyses suggest that the exacerbation of acne progression by stress may be associated with complex dysregulation of lipid metabolism and neuroregulatory processes, which may ultimately enhance inflammatory responses. Further analysis of gene expression associated with inflammation, lipid metabolism, and neuroregulation (Figure 4E) showed that, compared with controls, the genes related to inflammation were up-regulated in the Acne group. In the Acne_Pressure group, several inflammation- and lipid metabolism-related genes (e.g., *Cd86, Il36g, Stat3, Smpd3, Smpd1,* and *Dgat2*) showed significant changes, with lipid metabolism-related genes being particularly pronounced. Additionally, *Fasn* expression also showed an upward trend in the Acne_Pressure group and Pressure group. Genes in the neuro-related module, such as *Ngf, Ntf5,* and *Prlr*, also exhibited altered expression trends.

It has been reported that psychological stress triggers cortisol release via the HPA axis, which may impair skin barrier function, disrupt keratinocyte differentiation, increase susceptibility to skin infection, and contribute to the exacerbation of atopic dermatitis and delay in wound healing [2]. However, its role in the pathogenesis of acne remains poorly understood. Therefore, we investigated transcriptomic alterations in genes related to barrier function, including those involved in tight junction formation and keratinization. Expression of related genes (e.g., *Krt* family and *Dsg* family) was significantly altered in both Acne and Acne_Pressure groups, with more pronounced changes in the Acne_Pressure group (Figure 4D). Further pathway enrichment analysis of DEGs among the groups indicated that these genes were mainly enriched in inflammation, lipid metabolism, and immune regulation-related pathways (Figure 4F), suggesting that these pathways may play key roles in the mechanisms by which stress exacerbates acne pathophysiology. Overall, *C. acnes* infection combined with stress may contribute to acne progression by regulating the expression of genes related to cell junctions, keratinization, inflammatory responses, lipid metabolism, and neuroendocrine regulation.

## Discussion

As a chronic inflammatory skin disease, acne vulgaris involves inflammation throughout the course of the disease, causing irreversible damage to the skin (such as post-inflammatory hyperpigmentation and scarring) and significantly affecting patients’ quality of life. Numerous studies have identified psychological stress as a key risk factor that exacerbates acne severity. Stress and acne may interact via the ‘brain–skin axis,’ yet the specific molecular regulatory mechanisms remain unclear [3]. Focusing on the relationship between stress and acne progression and identifying the key mediators and functional pathways involved are crucial for developing therapeutic strategies that address both psychological and physiological aspects. Animal models are indispensable tools for achieving these research goals.

In the present study, we observed that the chronic restraint stress model rendered mice more susceptible to *C. acnes*-induced skin inflammatory responses. Chronic restraint stress is a classical model used to mimic anxiety- and depression-like states and has been widely applied in dermatological research [2]. Previous studies have shown that psychological stress can activate the HPA axis, promoting cortisol release and thereby affecting immune homeostasis and skin barrier function [19,20]. Our results confirmed that stress-exposed mice exhibited elevated corticosterone levels. When stress was combined with *C. acnes* infection, mice showed more pronounced epidermal thickening, inflammatory cell infiltration, and increased levels of inflammatory cytokines in both tissue and serum.

Moreover, prior research has demonstrated that keratinocytes, as one of the key host cells for *C. acnes*, play roles beyond forming a physical barrier—they actively participate in innate immune responses and inflammation regulation during acne pathophysiology [15,21]. In our study, *C. acnes* stimulation induced keratinocytes to release large amounts of pro-inflammatory factors such as IL-8 and TNF-α. Interestingly, with the addition of different concentrations of cortisol (to mimic stress), the levels of inflammatory factors decreased. This discrepancy between cellular and animal-level results indicates that corticosterone may not directly drive inflammatory progression but instead modulates acne progression through a series of complex regulatory pathways. How stress regulates the inflammatory microenvironment of acne via the ‘brain–skin axis’ thus remains to be further elucidated.

In the present study, transcriptomic analysis of ear tissues from the animal models identified changes that may reflect potential molecular pathways associated with stress-related exacerbation of acne-like inflammation. Compared with the Acne group, the Acne_Pressure group showed significant activation of lipid metabolism pathways. Previous studies have shown that dysregulated lipid metabolism can lead to increased sebum production, which is an important mechanism in acne development [22]. These transcriptomic findings support the notion that stress-induced alterations in the lipid profile may create a favorable environment for acne exacerbation.

Prior research has indicated that lipid metabolic reprogramming can affect inflammatory responses in the skin microenvironment, where abnormal accumulation of sphingolipids and triglycerides promotes keratinocyte dysregulation and pro-inflammatory factor release [23,24]. Our transcriptomic data showed that the PPAR signaling pathway was significantly enriched in the Acne_Pressure group and Pressure group. This nuclear receptor has been demonstrated to act as a bridge between sebum secretion and immune responses [25,26]. We speculate that stress may modulate lipid homeostasis via PPAR-mediated pathways to aggravate inflammatory lesions, but the current transcriptomic evidence supports this as an exploratory finding and requires future validation.

Fatty acid synthase (FASN) is the key rate-limiting enzyme in *de novo* fatty acid synthesis, and its overactivation can lead to accumulation of saturated fatty acids in sebum, promoting abnormal follicular keratinization [27]. Previous studies have confirmed that PPARγ can regulate FASN expression to disrupt sebaceous follicle unit homeostasis and contributes to local inflammatory microenvironment formation [28]. Abnormal lipid synthesis and secretion alter the composition of surface lipids in the skin, thereby impairing stratum corneum barrier function [29]. The transcriptomic data suggested a modest increase in *Fasn* expression in both the Acne_Pressure and Pressure groups, implying that lipid metabolism-related changes may be involved in the response to stress. Altered expression of *Smpd3* may indicate dysregulated sphingolipid metabolism and ceramide-related signaling, potentially contributing to impaired epidermal barrier homeostasis and inflammatory dysregulation in stress-associated acne-like inflammation [30,31]. Nevertheless, the present data do not establish a direct causal relationship between stress-induced lipid metabolic alterations, *C. acnes* colonization, and chronic inflammation. Further functional studies are needed to clarify these potential mechanisms.

Additionally, the changes in the expression of neuroendocrine-related genes in the Acne_Pressure group also highlight the complex interplay between stress and acne pathology. These preliminary analytical findings are consistent with the concept of the ‘brain–skin axis’. Neuroendocrine-related genes such as *Ntf5, Ngf, and Prlr* play a role in skin homeostasis and immune responses [32]. The dysregulation of neuroregulatory pathways and gene expression under the combined conditions of stress and acne suggests the potential of targeting the ‘brain–skin axis’ in novel therapeutic strategies, providing multidimensional diagnostic and treatment approaches for patients who are affected by stress. Overall, stress may modulate acne progression through multi-system interactions, and targeting neuroendocrine–metabolic–immune regulation may represent a new intervention strategy. The interaction between stress and inflammation further complicates the pathogenesis of acne, and interventions that modulate stress mediators may help alleviate stress-induced exacerbation of the disease.

Finally, our study found that stress may contribute to skin barrier dysfunction in acne. In the Acne_Pressure group, genes related to tight junction formation and keratinization, such as members of the *Krt*, *Dsg*, and *Cldn* families, showed significant changes in expression. Dysregulation of these genes may compromise the structural integrity of the skin barrier, increasing susceptibility to external irritants and pathogenic microorganisms, which can further trigger or exacerbate inflammatory responses.

Currently, therapeutic strategies for acne still mainly focus on conventional targets, encompassing antimicrobial, anti-inflammatory and sebum-regulating approaches. These include systemic or topical retinoids to normalize follicular keratinization, antibiotics to suppress the abnormal proliferation of *C. acnes* and control inflammatory progression, as well as oral contraceptives to modulate hormonal levels in female patients [33]. However, clinical outcomes are often suboptimal with high recurrence rates, and the adverse effects associated with long-term treatment frequently place patients in a therapeutic dilemma [34]. Based on the preliminary findings of the present study, future therapeutic development may shift toward multi-target interventions that simultaneously modulate neuroendocrine pathways, lipid metabolism, and inflammatory signaling. Recent advances in traditional Chinese medicine research have provided new therapeutic insights. Certain herbal medicines and their bioactive constituents have demonstrated unique advantages in modulating neuroendocrine activity and suppressing inflammatory responses, thereby offering novel strategies for the management of stress-related acne. For example, spinosin, a flavonoid component derived from *Ziziphi Spinosae Semen*, has been shown to exert sedative and anxiolytic effects, while flavonoids more broadly have demonstrated immunomodulatory properties in chronic inflammatory skin diseases [35,36].Total glycosides from *Cistanche tubulosa* can alleviate chronic stress-induced inflammation and intestinal barrier disruption by regulating HPA axis function and abundance of inflammation-associated gut microbiota [37]. Lipophilic diterpenoid compounds (tanshinones) isolated from *Salvia miltiorrhiza* have also shown therapeutic potential in acne, likely through anti-inflammatory and immunoregulatory mechanisms. Furthermore, above-ground parts of *Paris yunnanensis Franch* attenuate acne progression and promote tissue repair by inhibiting follicular duct dilation and reducing the production of perifollicular proinflammatory cytokines, including IL-6, IL-1β, and TNF-α [38]. Quercetin promotes skin barrier repair and mitigates post-inflammatory hyperpigmentation and scar formation through its antioxidative and antifibrotic effects [39]. Lycopene is expected to prevent and mitigate oxidative stress and chronic inflammation [40]. Based on the preliminary evidence for potential neuroendocrine–metabolic–immune regulatory networks obtained from the present study, future research may further investigate the multi-target advantages of traditional Chinese medicine formulas or isolated bioactive compounds.

There are several limitations in the present study. First, skin barrier dysfunction was inferred from transcriptomic changes in genes encoding structural proteins and tight junction components (e.g., Krt, Dsg, and Cldn family members); direct functional measurements of barrier integrity, such as transepidermal water loss, were not performed and should be included in future studies to confirm these findings. Second, the DEGs identified by RNA-seq were not independently validated by qRT-PCR in the present study; therefore, the transcriptomic findings should be interpreted with appropriate caution and will be confirmed in subsequent targeted studies. In addition, as the present study was conducted using a murine model, the translational relevance of our findings to human acne remains to be established, and transcriptomic pathway analyses should be interpreted as exploratory, and further functional validation studies are required to confirm the identified candidate pathways. Additionally, the present study used only male mice; given the documented sex differences in stress-induced inflammatory responses, future studies should include both sexes to evaluate potential sex-dependent differences in stress–acne interactions. Moreover, the *in vitro* experiments were performed exclusively using the HaCaT immortalized human keratinocyte cell line. Given that acne pathogenesis involves multiple cell types, including sebocytes and immune cells, future studies should incorporate additional relevant cellular components through co-culture systems or three-dimensional skin models to better mimic the *in vivo* microenvironment. Despite these limitations, the present study provides novel insights and experimental evidence into the molecular mechanisms by which stress exacerbates acne.

## Conclusion

In conclusion, our findings suggest that psychological stress may aggravate *C. acnes*-induced acne inflammation in mice, and this process may be associated with alterations in lipid metabolism, neuroendocrine regulation, immune responses, and abnormal skin barrier function. These transcriptomic alterations provide preliminary evidence supporting the possible involvement of neuroendocrine–metabolic–immune interactions in stress-associated acne exacerbation. Nevertheless, the present study does not establish direct causal mechanisms, and further functional validation is required to determine how these pathways contribute to acne progression under psychological stress. These findings may provide a basis for future investigations into multi-target strategies for the management of stress-related acne.

## Supplementary Material

Supplementary Figures S1-S3

## Data Availability

All other data are included in the manuscript and its supplementary files. The RNA-seq data generated in the present study have been deposited in the Gene Expression Omnibus (GEO) database under accession number GSE325321 [41].

## Abbreviations

ANOVA: analysis of variance
DEGs: differentially expressed genes
ELISA: enzyme-linked immunosorbent assay
FASN: fatty acid synthase
GO: Gene Ontology
H&E: hematoxylin–eosin
HPA: hypothalamic–pituitary–adrenal
