# Supplementary material for: Comprehensive analysis of phenotypes and transcriptome characteristics reveal important contribution of chronic stress and inflammation in the pathogenesis of acne
Source: Biosci Rep. 2026 Jul 24;46(8):BSR20260230. doi: 10.1042/BSR20260230 (PMC13402863; doi:10.1042/BSR20260230)
Supplement: Supplementary Figures S1-S3 [file BSR-2026-0230_supp.pdf]

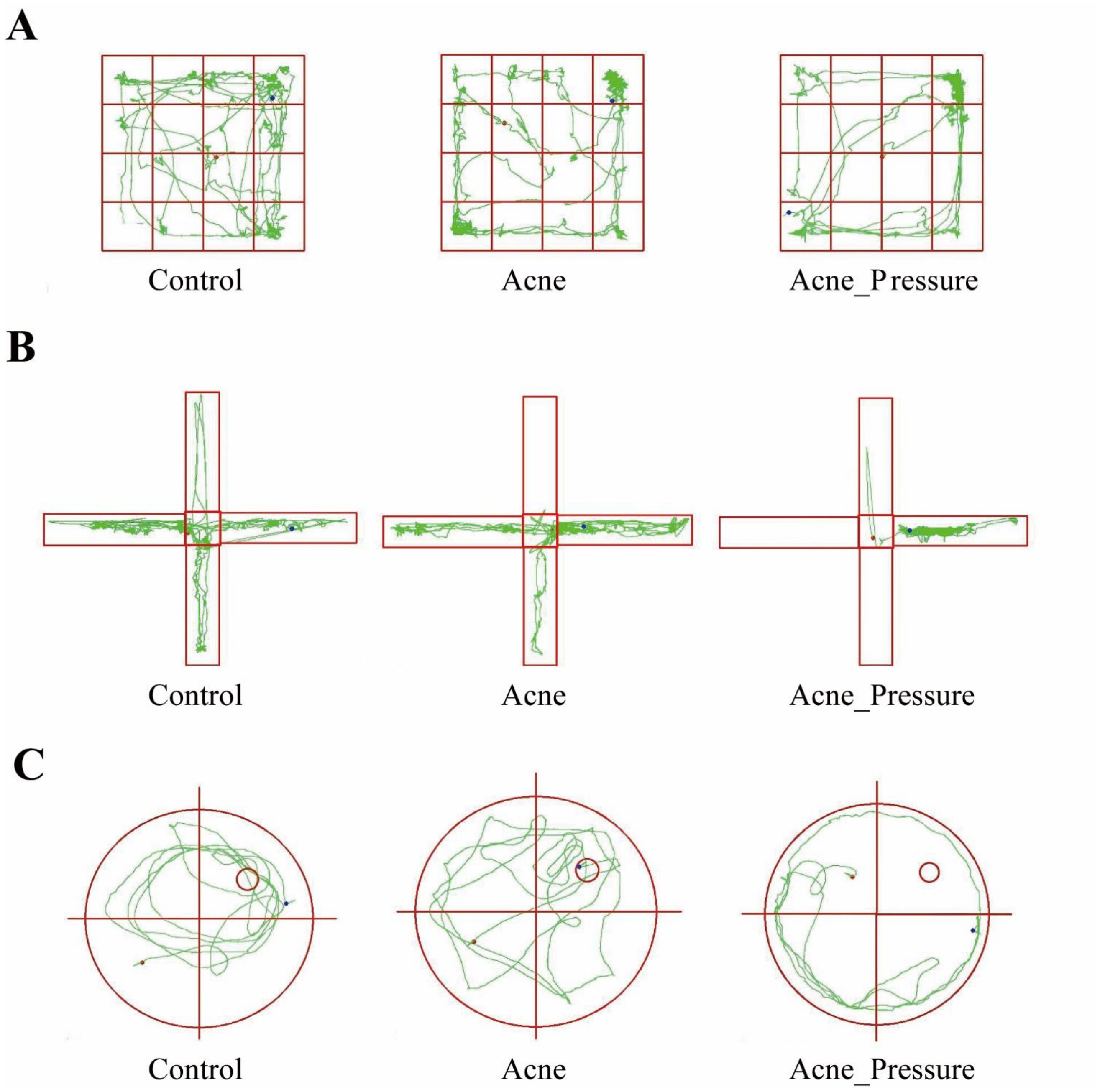

Supplementary Figure 1. Results of behavioral experiments

(A) Open field test – the less total distance traveled by the mice, the lower their activity; shorter time spent in the central zone and fewer entries indicate higher anxiety levels. **(B)** Elevated plus-maze test – fewer entries into open arms and shorter time spent there suggest more pronounced anxiety-like behavior. **(C)** Morris water maze test – longer escape latency indicates poorer learning and memory ability; lower percentage of time spent in the target quadrant and fewer platform crossings reflect impaired spatial memory.

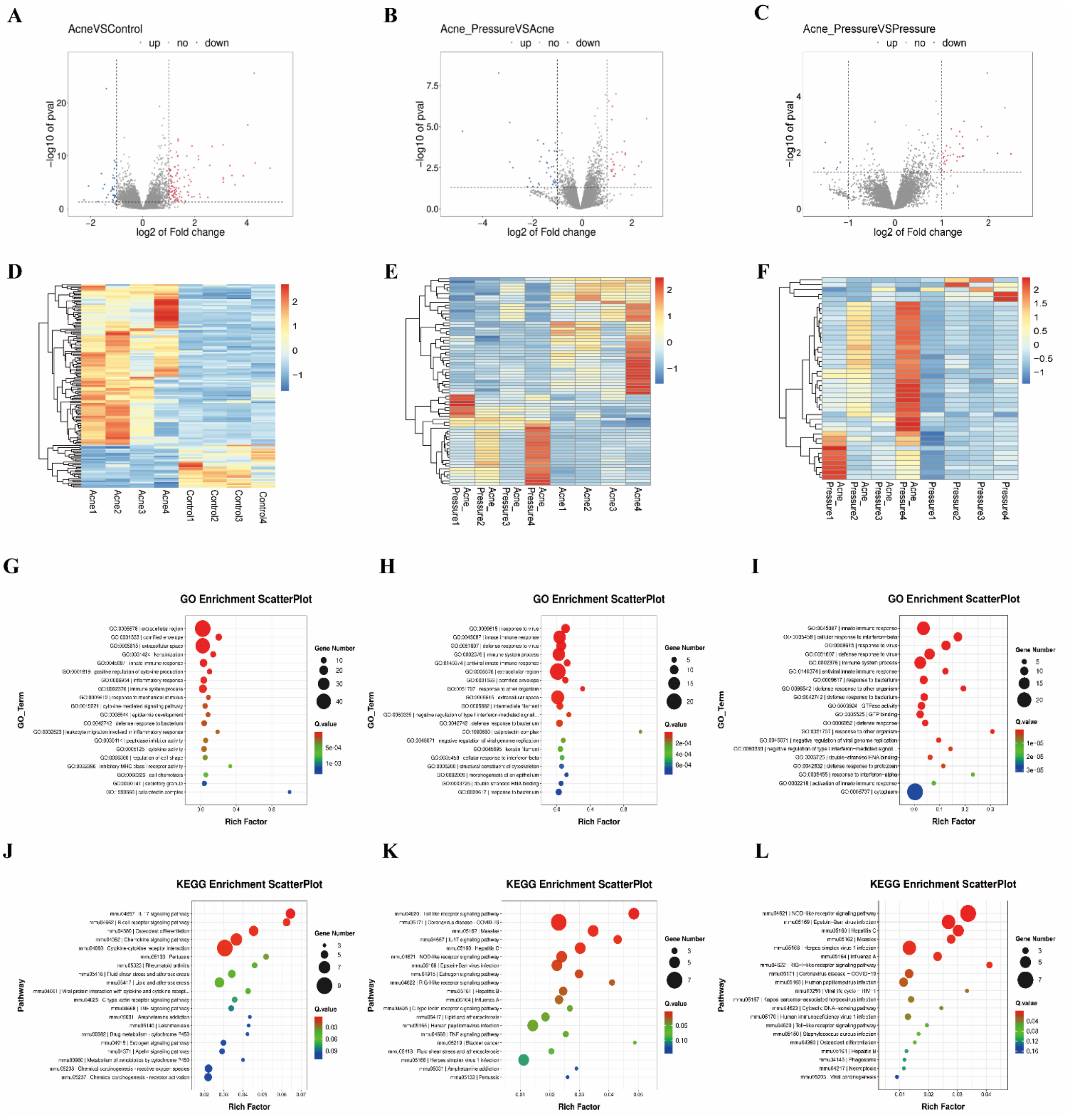

Supplementary Figure 2. Transcriptome enrichment results

(A) Volcano plot for Acne vs Control: red/blue dots indicate significantly upregulated/downregulated genes. **(B)** Volcano plot for Acne_Pressure vs Acne: red/blue dots indicate significantly upregulated/downregulated genes. **(C)** Volcano plot for Acne_Pressure vs Pressure: red/blue dots indicate significantly upregulated/downregulated genes. **(D)** Cluster heatmap for Acne vs Control: colors from red to blue represent gene expression levels from high to low. **(E)** Cluster heatmap for Acne_Pressure vs Acne: colors from red to blue represent gene expression levels from high to low. **(F)** Cluster heatmap for Acne_Pressure vs Pressure: colors from red to blue represent gene expression

levels from high to low. **(G)** GO enrichment plot for Acne vs Control: dot size represents the number of genes enriched in each function, colors from red to blue correspond to enrichment significance (smaller Q value indicates higher significance), and Rich Factor indicates the proportion of differential genes in each function. **(H)** GO enrichment plot for Acne_Pressure vs Acne: dot size represents the number of genes enriched in each function, colors from red to blue correspond to enrichment significance (smaller Q value indicates higher significance), and Rich Factor indicates the proportion of differential genes in each function. **(I)** GO enrichment plot for Acne_Pressure vs Pressure: dot size represents the number of genes enriched in each function, colors from red to blue correspond to enrichment significance (smaller Q value indicates higher significance), and Rich Factor indicates the proportion of differential genes in each function. **(J)** KEGG enrichment analysis for Acne vs Control. **(K)** KEGG enrichment analysis for Acne_Pressure vs Acne. **(L)** KEGG enrichment analysis for Acne_Pressure vs Pressure.

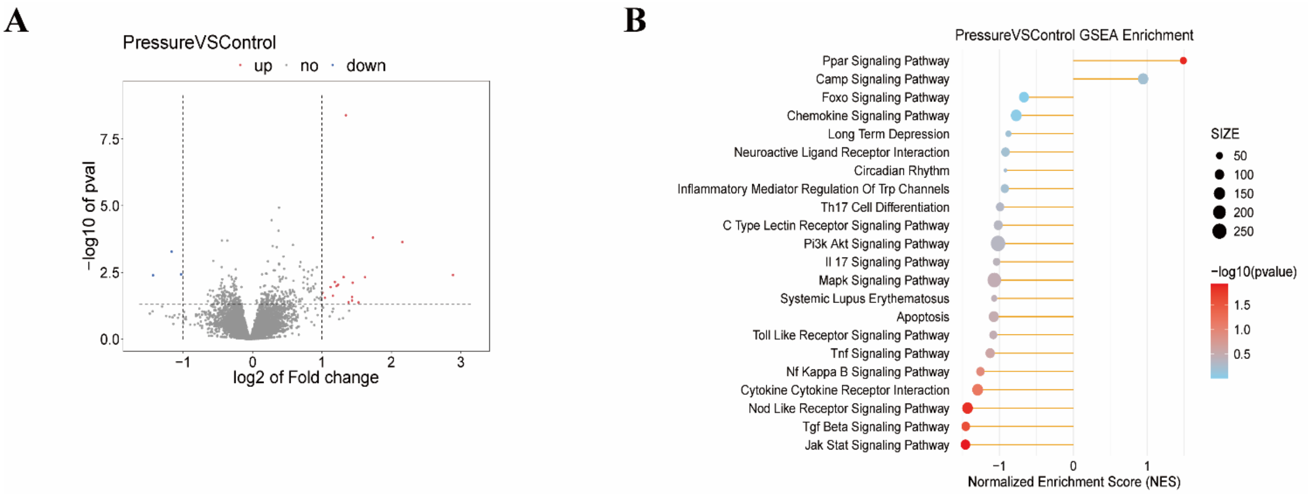

Supplementary Figure 3. Pressure vs. Control comparison in Transcriptomic analysis

(A) Volcano plot for Pressure vs Control: red/blue dots indicate significantly upregulated/downregulated genes. **(B)** GSEA pathway enrichment analysis for Pressure vs. Control (n = 4 per group).
